# The FEES Dysphagia Index: a bias-resilient continuous score that captures expert clinical judgment in 2,943 neurological inpatients

**DOI:** 10.1007/s00415-026-13895-x

**Published:** 2026-06-03

**Authors:** Cornelius J. Werner, Elizabet Sanchez-Garcia, Bettina Mall, Tareq Meyer, João Pinho, Joerg B. Schulz, Beate Schumann-Werner

**Affiliations:** 1 Department of Neurology, Medical Faculty, RWTH Aachen University, Aachen, Germany; 2 Department of Neurology and Geriatrics, Johanniter-Hospital Stendal, Stendal, Germany; 3 Department of Geriatric Medicine, Cork University Hospital, Cork, Ireland; 4 Institute of Cognitive Neurology and Dementia Research (IKND), Otto von Guericke University Magdeburg, Magdeburg, Germany

**Keywords:** Dysphagia, FEES, Selection bias, Penetration–Aspiration Scale, Clinical decision support

## Abstract

Multi-consistency testing during flexible endoscopic evaluation of swallowing (FEES) is clinically necessary, but introduces selection bias: worst scores inflate severity because the number of consistencies tested covaries with disease severity. In this retrospective observational study of hospitalized neurological patients, we derived and validated the FEES Dysphagia Index (FDI) in two temporally independent cohorts (Cohort 1: 2013–2018, *N* = 1,257; Cohort 2: 2021–2025, *N* = 1,686) from a single center. FDI-S averages Penetration–Aspiration Scale (PAS) scores across tested consistencies (0–100 scale); FDI-E uses Yale Pharyngeal Residue scores; FDI-C combines both. Selection bias was quantified using sequential branching-tree inverse probability weighting (IPW). Worst PAS overestimated severity by 24%; FDI deviated by < 2%. FDI-C was significantly superior to Worst PAS for hospital-acquired pneumonia (HAP; AUC 0.70 vs. 0.60, *p* < 0.001), mortality (0.71 vs. 0.62, *p* = 0.040), and restricted oral intake (0.90 vs. 0.74, *p* < 0.001), and statistically equivalent to clinician-rated severity. FDI-C mapped linearly onto ordinal Functional Oral Intake Scale values (FOIS; proportional odds RCS *p* = 0.99). With functional status and diagnosis, FDI-C reconstructed the clinician's oral intake recommendation with AUC up to 0.93. The FDI-C-mortality relationship was sigmoidal with a clinically relevant transition zone between ~ 50 and ~ 85. FDI-C is a bias-resilient, bedside-calculable score with interval-scale properties that captures expert clinical judgment, suitable as both a clinical decision support tool and a continuous research endpoint.

**Supplementary Information:**

The online version contains supplementary material available at 10.1007/s00415-026-13895-x.

## Introduction

Flexible endoscopic evaluation of swallowing (FEES) is a cornerstone of dysphagia assessment in neurological patients. Since its introduction by Langmore and colleagues [1], FEES has evolved into a widely available, bedside-compatible procedure that allows direct visualization of pharyngeal swallowing physiology without radiation exposure [2]. Current guidelines endorse FEES as a first-line instrumental assessment in acute stroke, neurodegenerative disease, and critical care settings [3–5].

The Penetration–Aspiration Scale (PAS) [6] remains the most widely used outcome measure derived from FEES. However, both its psychometric properties and its application in research remain contentious. Steele and Grace-Martin [7] demonstrated that the PAS does not function as a truly ordinal scale, with non-equidistant intervals and rarely observed levels, and proposed a physiologically motivated reclassification. In a comprehensive systematic review, Borders and Brates [8] documented that 183 studies using the PAS employed at least ten different categorization schemes, with no consensus on whether the scale should be treated as ordinal, categorical, or interval. Critically, among studies that administered multiple bolus types or consistencies, 55% reported PAS results only at the participant or group level, typically as a worst score, discarding information about consistency-specific patterns.

In routine practice, clinicians adapt consistency selection to the patient's presentation, omitting consistencies deemed unsafe or unnecessary—a process we term "clinical gating", in analogy to adaptive testing protocols in psychometrics where subsequent items are administered conditional on prior responses. Multi-consistency testing is clinically necessary, as bolus consistency is a primary determinant of penetration-aspiration risk: Borders and Steele [9], using Bayesian multilevel ordinal regression on two large videofluoroscopic datasets (n = 678 and n = 177, totaling over 11,500 bolus-level observations), demonstrated that thin liquids carry substantially higher PAS scores than thicker consistencies. However, multi-consistency testing introduces a methodological problem that has received little formal attention so far: number and type of consistencies tested will necessarily covary with disease severity, and therefore any summary statistic computed over a variable number of observations is biased when the number of observations depends on the outcome. Additionally, a survey of German FEES centers confirmed substantial variation in which consistencies are administered in the first place, further compounding this problem at a higher level in terms of comparing outcomes across different centers [10].

The consequences are well documented, but unresolved. Borders and Steele [9] stated explicitly that "the common practice of summarizing PAS scores based on worst scores is a bias." An additional Monte Carlo simulation by Borders and colleagues [11] demonstrated that worst-score aggregation of PAS data reduces statistical power and inflates effect size estimates compared to multilevel approaches that use all available bolus trials. Yet, no study has quantified the magnitude of this bias in the context of variable consistency testing of actual patients, and no scoring method has been proposed that addresses it.

The same problem affects existing composite scores. The Dynamic Imaging Grade of Swallowing Toxicity adapted for FEES (DIGEST-FEES) [12, 13] uses the maximum PAS as its entry point for safety grading and the maximum pharyngeal residue for efficiency grading. The Fiberoptic Endoscopic Dysphagia Severity Scale (FEDSS) [14, 15] assigns severity based on the worst finding across a sequential testing protocol. The ordinal FEES dysphagia score [16], a 4-level classification grading dysphagia from none through mild (premature spillage and/or residue without penetration/aspiration) to severe (penetration/aspiration of two or more consistencies), was proposed in the context of the integrated FEES report [4] and has been applied in studies of deep brain stimulation, myasthenia gravis, and Guillain-Barré syndrome [17–19]; it, too, relies on worst-finding logic across consistencies. Neither score adjusts for the variable number of consistencies administered, and both therefore inherit the structural vulnerability to clinical gating.

The primary objective of this study is to derive and temporally validate the FEES Dysphagia Index (FDI), a bias-resilient continuous score that corrects for clinical gating in multi-consistency FEES testing. Specifically, we derive FDI-S in a 2013–2018 cohort (*N* = 1,257) and replicate its bias resilience in an independent 2021–2025 cohort (*N* = 1,686) from the same center. Secondary objectives are (i) to quantify the selection bias introduced by clinical gating using sequential inverse probability weighting as a formal reference; (ii) to extend the framework to swallowing efficiency (FDI-E, based on Yale Pharyngeal Residue scores [20]) and a combined composite (FDI-C); (iii) to evaluate whether FDI-C captures the swallowing physiology component of clinical decision-making and its complementarity with functional status and diagnostic context, including its correspondence with clinician-assigned oral intake levels; and (iv) to assess FDI-C's suitability as both a clinical decision support tool and a continuous endpoint for dysphagia research.

## Methods

### Study design

This retrospective two-cohort observational study analyzed consecutive neurological inpatients who underwent FEES at the Department of Neurology, RWTH Aachen University Hospital. Although the analysis was designed retrospectively, the core clinical variables (PAS per consistency, Yale Pharyngeal Residue scores, FOIS, and FEES severity summaries) were documented prospectively at the time of examination as part of a standardized clinical FEES protocol, which limits information bias and preserves consistent variable definitions across the observation period. Reporting follows the STROBE guidelines for observational studies (Online Resource 8).

Cohort 1 (derivation) comprised all FEES patients from January 2013 to December 2018 (*N* = 1,257). Cohort 2 (replication and extension) comprised all FEES patients from January 2021 to December 2025 (*N* = 1,686 of 18,851 total inpatient episodes). The two cohorts do not overlap. The 2019–2020 period was excluded by design to avoid atypical referral patterns, infection-control-driven restrictions on FEES testing, and modified clinical workflows during the COVID-19 pandemic.

FEES was performed as part of routine clinical care according to a standardized protocol [2, 4].

The study was approved by the ethics committee of RWTH Aachen University (EK 088/17) and was performed in accordance with the ethical standards laid down in the 1964 Declaration of Helsinki and its later amendments. Individual informed consent was waived by the ethics committee due to the retrospective design and the use of routinely collected clinical data.

### FEES protocol

FEES examinations followed a sequential multi-consistency protocol. Up to four consistencies were tested: puree, thin liquid, thickened liquid (if deemed necessary after liquid), and solid food, in this order. Consistency selection followed clinical judgment: examiners could omit or add consistencies based on the patient's clinical presentation and observed swallowing performance (clinical gating). For each tested consistency, the Penetration–Aspiration Scale (PAS; 1–8) [6] was documented. In Cohort 2, the Yale Pharyngeal Residue Severity Rating Scale (1–5, separately for valleculae and pyriform sinuses) [20] was additionally documented as part of enhanced routine documentation.

### FDI construction

**FDI-S (Safety).** The arithmetic mean of PAS scores across all tested consistencies, linearly rescaled to 0–100 (100 = no impairment):

$${\mathrm{FDI}} - {\text{S }} = \, \left( {{1 } - \, \left( {{\text{mean PAS }} - { 1}} \right) \, /{ 7}} \right) \, \times { 1}00.$$

This formulation averages rather than maximizes, thereby normalizing for the variable number of consistencies tested. Unlike the Worst PAS, whose mathematically expected value increases with the number of observations, the arithmetic mean is unbiased with respect to the number of consistencies, provided the tested consistencies are representative of the untested ones conditional on observed covariates.

**FDI-E (Efficiency; Cohort 2 only).** The arithmetic mean of Yale combined scores (mean of valleculae and pyriform sinus scores) across tested consistencies, rescaled to 0–100:

$${\mathrm{FDI}} - {\text{E }} = \, \left( {{1 } - \, \left( {{\text{mean Yale combined }} - { 1}} \right) \, /{ 4}} \right) \, \times { 1}00.$$

**FDI composite (FDI-C; Cohort 2 only).** FDI-C = (FDI-S + FDI-E)/2, giving equal weight to safety and efficiency. The equal-weight specification was chosen a priori to avoid outcome-specific optimization and to maximize clinical interpretability. A sensitivity analysis examining regression-derived and AUC-maximizing alternative weightings of FDI-S and FDI-E confirmed that equal weighting performs comparably across all outcomes (Online Resource 3). A parallel sensitivity analysis for the valleculae vs. pyriform sinus weighting within FDI-E confirmed that the simple Yale mean generalizes across consistencies better than regression-derived empirical weights, which reverse direction between consistencies (Online Resource 7).

### Comparator scores

**Worst PAS.** The maximum PAS across all tested consistencies [6, 8].

**Ordinal FEES severity score (Cohort 2).** A four-level ordinal classification (0 = none, 1 = mild, 2 = moderate, 3 = severe) based on the combination of premature spillage, penetration-aspiration, and residue thresholds across tested consistencies [4, 16]. This classification uses a maximum-based logic, assigning severity based on the worst findings.

**Clinician-rated severity (Cohort 2).** A seven-level ordinal severity grading (0, 0.5, 1, 1.5, 2, 2.5, 3) with 0 = no dysphagia to 3 = severe dysphagia, extracted from the FEES summary text and converted to numerical values, capturing the clinician's holistic severity judgment. Half-steps (e.g., 1.5 for "mild-to-moderate") accommodate composite gradings used in clinical practice.

### Clinical outcomes

**Cohort 1:** Hospital-acquired pneumonia (HAP), defined as ICD-10 J69 coded as any-position diagnosis during the inpatient stay and used as a pragmatic proxy for aspiration-related pneumonia given that J69 may also capture chemical pneumonitis (12.7%); in-hospital mortality (3.3%); and prolonged length of stay (> median 15 days, 48.8%; Online Resource 2).

**Cohort 2:** HAP (ICD-10 J69 as a secondary diagnosis, 14.9%), in-hospital mortality (4.3%), and restricted oral intake (FOIS ≤ 3, 24.2%).

### Inverse probability weighting

To quantify selection bias, we constructed inverse probability weights (IPW) using a sequential branching-tree model that mirrored the clinical testing decisions [21]. At each decision node (puree → liquid, liquid → solid, liquid → thickened liquid), a logistic propensity model included baseline covariates (age, sex, Hospital Frailty Risk Score [22], Self-Care Index at admission [23, 24], neurological diagnosis) and PAS scores from preceding consistencies; in Cohort 2, Yale residue scores were added where likelihood ratio tests confirmed their predictive relevance. Full propensity-model specification, branch-by-branch Yale-covariate justification, propensity-score truncation, and stabilized-weight diagnostics (including the positivity violation in the thickened-liquid branch) are reported in Online Resource 1.

### Bias quantification

We used a three-cohort design: (1) the complete-case subcohort (all four consistencies tested), (2) the standard-consistency subcohort (puree + liquid), and (3) the IPW-adjusted full cohort. The primary bias metric was the **Naive-IPW Delta**: |naive mean − IPW mean|/IPW mean × 100. A Naive-IPW Delta below 1% indicates that the naive FDI already approximates the causal estimand. The conditional-independence assumption underlying both IPW and FDI, the direction of any residual bias, and the implications of clinical gating being potentially informative (MNAR) are discussed in detail in **Online Resource 9**.

### Predictive validity

Discrimination was assessed using AUC with DeLong comparisons [25]. Significance was set at α = 0.05 (two-sided). No correction for multiple comparisons was applied to DeLong tests, consistent with the exploratory nature of pairwise score comparisons; individual p-values should be interpreted accordingly.

### Scale properties (Cohort 2)

Existing FEES scores are ordinal at best: DIGEST-FEES yields five levels, FEDSS 6, and the ordinal FEES severity score 4. Because ordinal scales limit the choice of statistical methods and reduce power in clinical trials [26, 27], we assessed whether FDI-C meets the empirical criteria for interval-scale treatment along five dimensions: granularity (number of unique values), linearity of the logit (restricted cubic splines), information loss (AIC, continuous vs. quintile models), calibration (observed outcome rates across deciles), and construct validity against ordinal FOIS (Spearman correlation and proportional-odds regression). Full methodological detail—RCS knot specification, AIC comparison protocol, and the proportional-odds construct-validity model—is reported in Online Resource 10.

### Clinical decision model (Cohort 2)

In clinical practice, the FEES examiner determines swallowing severity and then contextualizes it within the patient's functional status and clinical acuity to recommend an oral intake level (FOIS). A patient with moderate dysphagia but preserved independence and chronic adaptation will receive a more liberal diet than a patient with similar swallowing impairment who is acutely ill, frail, and lacks established protective mechanisms. We formalized this clinical reasoning in a statistical model:

$${\text{FOIS }}\sim {\text{ FDI}} - {\text{C }} + {\text{ SPI }} + {\text{ diagnosis}},$$

where FDI-C captures swallowing physiology, SPI captures functional status, and neurological diagnosis (stroke, neurodegenerative, other) serves as a proxy for acuity and chronicity. We fitted (A) a proportional odds model for ordinal FOIS (1–7), and (B) logistic models for binary FOIS ≤ 3 and HAP, using nested model comparison (M1: FDI-C alone → M2: + SPI → M3: + diagnosis). Incremental contributions were assessed by likelihood ratio tests and DeLong AUC comparisons.

For mortality (*n* = 42 events in the complete-case subsample), the three-predictor model was not fitted because the events-per-variable ratio (EPV = 42/4 = 10.5) falls at the threshold below which logistic regression estimates become unreliable [28] The FDI-C–mortality relationship was instead characterized descriptively using restricted cubic splines.

### Inter-rater reliability

Because FDI is computed deterministically from PAS and Yale ratings without additional rater-dependent judgments, its composite IRR was estimated using the Spearman–Brown prophecy formula [29] from published single-consistency IRR values: ρ₁ = 0.85 for PAS [8, 30] and ρ₁ = 0.751 for Yale [20, 31]. The formula, worked examples for k = 2–4 consistencies, and the boundedness derivation for FDI-C (PAS IRR exceeds Yale IRR; FDI-C reliability is bounded from below by FDI-E) are presented in Online Resource 11.

### Internal validation (Cohort 2)

FDI-S was validated by temporal replication across cohorts (see *Study design*). Because FDI-E and FDI-C are novel to Cohort 2, their predictive performance required independent confirmation. We assessed stability in a stratified 60/40 random split (derivation *n* = 1,011, validation *n* = 675), computing AUCs for all four outcomes in each split and comparing them using DeLong tests. The three-cohort bias analysis was repeated within each split to confirm that bias resilience was not dependent on the specific sample composition. Stability thresholds and the full split AUCs are reported in Online Resource 4.

### Statistical analysis

All analyses were performed in R version 4.5.2 [32]. Full package list, including pROC, rms, MASS, cobalt, and the ordinal package used for Cohort 1 cumulative link mixed models, is provided in Online Resource 12.

## Results

### Cohort characteristics

The combined sample comprised 2,943 neurological inpatients. Cohort 1 (*N* = 1,257) had a mean age of 71.6 years (SD 13.7; median 74, IQR 64–82), was 55.0% male, and included 50.0% stroke, 22.3% neurodegenerative, and 27.7% other diagnoses. Hospital-acquired pneumonia (HAP) occurred in 160 patients (12.7%) and mortality in 42 (3.3%). Cohort 2 (*N* = 1,686) included 57.1% stroke, 19.2% neurodegenerative, and 23.7% other diagnoses. HAP occurred in 251 (14.9%), mortality in 73 (4.3%), and restricted oral intake (FOIS ≤ 3) in 392 (24.2%). Sixty-three patients in Cohort 2 (3.7%) had no valid FOIS documented and were treated as missing for FOIS-dependent analyses. Chart review identified three groups: failed or aborted examinations (*n* = 26), complete assessments with narrative documentation but missing structured fields (*n* = 24), and complete assessments with FDI scores where FOIS was simply not recorded (*n* = 13). None represented a distinct clinical state; the missing rate (3.7% overall, 0.8% among patients with calculable FDI) is unlikely to introduce systematic bias. Full characteristics are presented in Table 1.

**Table 1:** Patient characteristics

Characteristics	Cohort 1 (2013–2018, *N* = 1,257)	Cohort 2 (2021–2025, *N* = 1,686)
Age, years, mean (SD)	71.6 (13.7)	70.6 (13.7)
Male sex, n (%)	691 (55.0)	950 (56.3)
Neurological diagnosis		
Stroke	629 (50.0)	963 (57.1)
Neurodegenerative disease	280 (22.3)	324 (19.2)
Other neurological	348 (27.7)	399 (23.7)
Functional status		
SPI at admission, mean (SD)	26.0 (10.5)^a^	27.7 (9.4)
HFRS, mean (SD)	11.0 (6.2)	11.4 (6.6)
Outcomes		
HAP (J69)	160 (12.7)	251 (14.9)
In-hospital mortality	42 (3.3)	73 (4.3)
FOIS ≤ 3	n/a	392 (24.2)^b^
Prolonged LOS^c^	613 (48.8)	836 (49.6)
LOS, median (IQR), days	15 (9–21)	16 (8–23)

*FOIS* Functional Oral Intake Scale, *HFRS* Hospital Frailty Risk Score, *IQR* interquartile range, *J69* ICD-10 code for pneumonitis due to solids and liquids, *LOS* length of stay, *n* number, *SD* standard deviation, *SPI* Self-Care Index

^a^n = 1,125 with SPI available

^b^Excludes 63 patients with missing/invalid FOIS

^c^ > median (Cohort 1: > 15 d; Cohort 2: > 16 d). LOS in Online Resource 2

### Consistency testing patterns

Both cohorts showed the expected clinical gating pattern. In Cohort 1, puree and thin liquid were tested in > 91% of patients, whereas thickened liquid and solid were tested in 43.8% and 63.4%, respectively; only 331 patients (26.3%) received all four consistencies. In Cohort 2, testing rates were similar: puree 91.3%, liquid 89.0%, solid 68.0%, thickened liquid 35.3%; complete cases *n* = 412 (24.4%) (Table 2). The consistency of these patterns across two cohorts separated by a temporal gap confirms that clinical gating is a stable institutional practice, not a sampling artifact.

**Table 2:** Consistency testing patterns and FDI score distributions

Panel A: Consistencies tested		
Consistency	Cohort 1 (*N* = 1,257)	Cohort 2 (*N* = 1,686)
Puree	1,145 (91.1)	1,539 (91.3)
Thin liquid	1,156 (92.0)	1,500 (89.0)
Solid	797 (63.4)	1,147 (68.0)
Thickened liquid	550 (43.8)	595 (35.3)

Panel B: Testing pathways		
No. of consistencies	Cohort 1	Cohort 2
1	113 (9.0)	51 (3.0)
2	228 (18.1)	175 (10.4)
3	585 (46.5)	893 (53.0)
4 (all)	331 (26.3)	412 (24.4)

Panel C: Score distributions (Cohort 2)					
Score	n	Mean (SD)	Median	Range	Unique values
FDI-S	1,559	72.6 (25.7)	75.0	0–100	40
FDI-E	1,557	64.5 (21.3)	66.7	0–100	45
FDI-C	1,557	68.6 (20.6)	71.1	0–100	387
Worst PAS	1,559	4.9 (2.8)	5.0	1–8	8
Ordinal FEES severity	1,686	2.1 (0.9)	–	0–3	4
Clinician-rated severity	1,535	1.7 (0.9)	–	0–3	7

Panel D: Score distributions (Cohort 1, FDI-S only)					
Score	n	Mean (SD)	Median	Range	Unique values
FDI-S	1,257	70.2 (30.6)	75.0	0–100	41
Worst PAS	1,257	4.6 (2.8)	5.0	1–8	8

*FDI-C* FEES Dysphagia Index composite, *FDI-E* FEES Dysphagia Index-Efficiency, *FDI-S* FEES Dysphagia Index-Safety, *FEES* flexible endoscopic evaluation of swallowing, *n* number, *PAS* Penetration–Aspiration Scale, *SD* standard deviation

### Consistency-specific safety profiles (Cohort 1)

The aspiration risk hierarchy was consistent across cohorts. In Cohort 1, thin liquid carried the highest risk (mean PAS 4.33; 36.0% aspiration), followed by thickened liquid (3.71; 26.2%), puree (2.26; 11.3%), and solid (1.36; 1.9%). In CLMMs, thin liquid (β = 2.087, *p* < 0.001) and thickened liquid (β = 1.186, *p* < 0.001) were associated with worse PAS relative to puree, while solid was associated with better scores (β =  − 1.339, *p* < 0.001); these effects were robust to adjustment for age, sex, HFRS, and SPI. Cohort 2 confirmed this hierarchy: thin liquid (mean PAS 4.77; 48.0% aspiration) > thickened liquid (3.52; 20.8%) > puree (1.93; 7.7%) > solid (1.16; 1.2%). The hierarchy was unchanged after IPW adjustment in Cohort 1, confirming that it reflects genuine consistency-specific risk rather than a selection artifact.

Inter-consistency PAS correlations were moderate in both cohorts (Cohort 1: Spearman ρ = 0.14–0.33; Cohort 2: ρ = 0.13–0.33), confirming that swallowing safety with one consistency does not reliably predict safety with others and providing empirical support for multi-consistency testing. Yale residue correlations in Cohort 2 were higher (ρ = 0.38–0.60), consistent with pharyngeal residue being a more stable trait across consistencies. Full pairwise correlation matrices for PAS and Yale across both cohorts are provided in Online Resource 5.

### Bias resilience

**Cohort 1 (derivation).** Worst PAS was inflated by 24.1% in the complete-case subcohort (i.e., patients tested on all four consistencies; mean 6.28 vs. IPW reference 5.06) and underestimated by 10.4% in the standard-consistency subcohort (restricted to puree and liquid only; mean 4.53). FDI-S deviated by < 2% from the IPW reference in both subcohorts (complete case: 67.15 vs. 68.43, bias + 1.9%; standard consistency: 67.77 vs. 68.43, bias + 1.0%). IPW correction changed FDI-S AUCs by < 0.01.

**Cohort 2 (replication and extension).** The Naive-IPW Delta was 0.5% for FDI-S, 0.6% for FDI-E, and 0.6% for FDI-C. Worst PAS showed a complete-case bias of 9.8% (complete-case mean 7.02 vs. IPW 6.39). This illustrates a well-known property of order statistics: the expected value of the maximum of a set of random variables increases with the number of observations, even when the underlying distribution is unchanged [33]. The complete-case subcohort consists of patients who were mild enough to proceed through all four testing stages (confirmed by their lower FDI-S), yet their Worst PAS is paradoxically inflated because having four rather than two or three tested consistencies provides more opportunities for an extreme PAS value on any single trial.

We performed a sensitivity analysis to test whether FDI's bias resilience depends on the specific IPW model. The thickened liquid branch had unstable weights (positivity violation at 35% testing rate), so we re-ran the IPW excluding this branch entirely. This changed the IPW reference values substantially (e.g., FDI-S shifted by 8.4 points between the two IPW specifications), indicating that the exact IPW-corrected population mean is sensitive to modeling choices. However, the Naive-IPW Delta—the discrepancy between the simple, unweighted FDI mean and the IPW-corrected mean—remained below 1% under both specifications. This means that regardless of which IPW model is used as the reference, the naive FDI consistently lands close to it. The practical implication is that FDI does not require IPW correction: the simple bedside-calculated mean is already a good approximation of the bias-corrected value. Detailed three-cohort bias results under both IPW specifications (full model and excluding the thickened liquid branch) are reported in Online Resource 6.

**Fig. 1:**
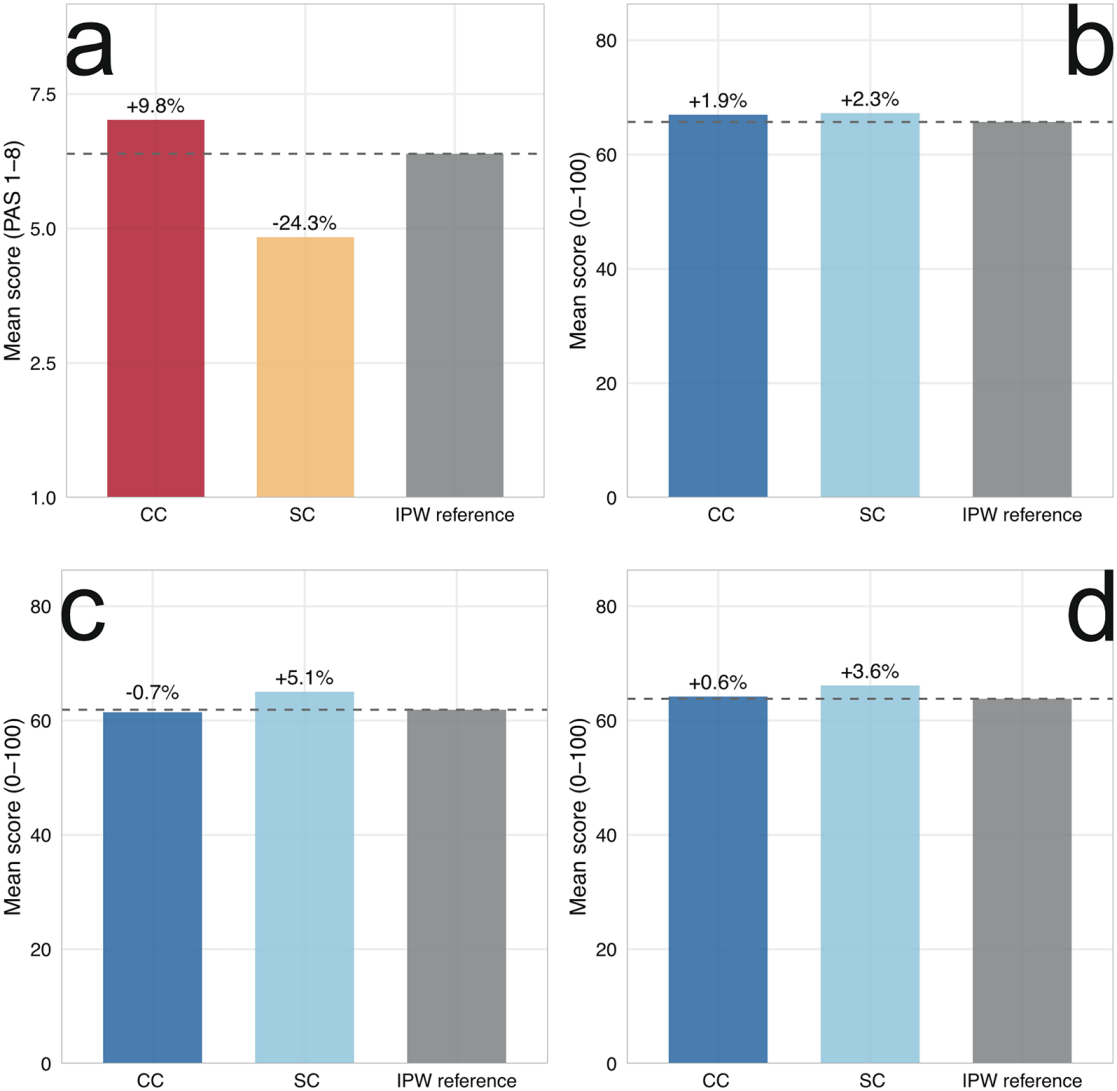
Three-cohort bias comparison for Cohort 2. Mean scores for the complete-case subcohort (all four consistencies tested, *n* = 412), the standard-consistency subcohort (puree + liquid, *n* = 1,480), and the IPW-adjusted reference (*n* = 1,559). **a** Worst PAS (1–8 scale). **b-d** FDI variants (0–100 scale), with b = FDI-S, c = FDI-E and d = FDI-C. Percentage labels indicate complete-case (CC) bias and standard-consistency (SC) bias relative to the IPW reference. *Abbreviations:* CC, complete case (all 4 consistencies tested); FDI-C, FEES Dysphagia Index composite; FDI-E, FEES Dysphagia Index-Efficiency; FDI-S, FEES Dysphagia Index-Safety; IPW, inverse probability weighting; n, number; PAS, Penetration–Aspiration Scale; SCD, standard consistency design (puree + liquid)

Results are presented in Table 3 and Fig. 1.

**Table 3:** Bias resilience: three-cohort analysis

Panel A: Cohort 1 (derivation)					
Score	CC mean (*n* = 331)	SCD mean (*n* = 1,062)	IPW ref (*N* = 1,257)	CC bias (%)	SCD bias (%)
Worst PAS	6.28	4.53	5.06	+ 24.1	− 10.4
FDI-S	67.15	67.77	68.43	− 1.9	− 1.0

Panel B: Cohort 2 (replication)					
Score	CC mean (*n* = 412)	SCD mean (*n* = 1,480)	IPW ref (*n* = 1,559)	CC bias (%)	Naive-IPW Δ (%)
Worst PAS	7.02	4.84	6.39	+ 9.8	n/a
FDI-S	66.97	67.24	65.70	+ 1.9	0.5
FDI-E	61.45	65.04	61.90	− 0.7	0.6
FDI-C	64.21	66.14	63.81	+ 0.6	0.6

Panel C: Sensitivity (IPW without thickened liquid, Cohort 2)				
Score	IPW ref (full)	IPW ref (no thick)	Δ references	Naive-IPW Δ
FDI-S	65.70	74.13	+ 8.43	0.5
FDI-C	63.81	69.73	+ 5.92	0.6

IPW diagnostics: mean 1.00 (SD 0.87), median 0.61, range 0.20–5.45

*CC* complete case (all 4 consistencies tested), *FDI-C* FEES Dysphagia Index composite, *FDI-E* FEES Dysphagia Index-Efficiency, *FDI-S* FEES Dysphagia Index-Safety, *IPW* inverse probability weighting, *n* number, *Naive-IPW Delta* |naive mean − IPW mean|/IPW mean × 100, *PAS* Penetration–Aspiration Scale, *SCD* standard consistency design (puree + liquid), *SD* standard deviation

### Predictive validity

**Cohort 1.** FDI-S consistently outperformed Worst PAS: HAP (AUC 0.712 vs. 0.644, *p* < 0.001), mortality (0.639 vs. 0.600, *p* = 0.020), and prolonged LOS (0.584 vs. 0.551, *p* < 0.001).

**Cohort 2.** FDI-C was significantly superior to Worst PAS for all outcomes: HAP (0.698 vs. 0.604, ΔAUC =  + 0.094, *p* < 0.001), mortality (0.706 vs. 0.622, + 0.083, *p* = 0.040), and FOIS ≤ 3 (0.900 vs. 0.735, + 0.164, *p* < 0.001). FDI-C was also significantly superior to the ordinal FEES severity score for HAP (*p* = 0.001) and FOIS ≤ 3 (*p* < 0.001).

FDI-C was statistically equivalent to clinician-rated severity for all outcomes (all *p* ≥ 0.148). For HAP, FDI-C achieved AUC 0.698 versus 0.707 for clinician-rated severity (*p* = 0.731). For FOIS ≤ 3, clinician-rated severity reached 0.916, numerically exceeding FDI-C (0.900), but the difference was not significant (*p* = 0.201). FDI-C correlated more strongly with clinician-rated severity (ρ =  − 0.829) than the ordinal FEES severity score did (ρ = 0.611 with clinician-rated severity), suggesting that a simple arithmetic mean captures the holistic assessment that experienced clinicians perform intuitively.

IPW correction changed AUC values by ≤ 0.008 for all FDI variants, confirming that bias resilience at the score level translates to the inferential level.

Results are presented in Table 4. Prolonged LOS results are reported in Online Resource 2.

**Table 4:** Predictive validity: AUC comparison

Panel A: Cohort 1 (FDI-S only)		
Score	Asp. pneumonia	Mortality
FDI-S	0.712 (0.671–0.754)	0.639 (0.553–0.724)
Worst PAS	0.644 (0.604–0.683)	0.600 (0.521–0.679)
DeLong (FDI-S vs Worst PAS)	+ 0.069, *p* < 0.001	+ 0.039, *p* = 0.020

Panel B: Cohort 2 (extended comparators)			
Score	Asp. pneumonia	Mortality	FOIS ≤ 3
FDI-C	0.698 (0.661–0.736)	0.706 (0.649–0.763)	0.900 (0.881–0.919)
FDI-S	0.690 (0.652–0.728)	0.692 (0.634–0.750)	0.899 (0.880–0.919)
FDI-E	0.656 (0.618–0.695)	0.672 (0.612–0.731)	0.777 (0.749–0.804)
Worst PAS	0.604 (0.569–0.640)	0.622 (0.567–0.678)	0.735 (0.710–0.760)
Ordinal FEES sev	0.611 (0.574–0.648)	0.619 (0.558–0.680)	0.721 (0.694–0.748)
Clinician-rated sev	0.707 (0.675–0.740)	0.760 (0.713–0.807)	0.916 (0.900–0.931)

Panel C: DeLong comparisons, FDI-C vs comparators (Cohort 2)			
Comparison	Asp. pneumonia	Mortality	FOIS ≤ 3
vs Worst PAS	+ 0.094, *p* < 0.001	+ 0.083, *p* = 0.040	+ 0.164, *p* < 0.001
vs Ordinal FEES sev	+ 0.087, *p* = 0.001	+ 0.087, *p* = 0.042	+ 0.178, *p* < 0.001
vs Clin. severity	− 0.009, *p* = 0.731	− 0.055, *p* = 0.148	− 0.016, *p* = 0.201

*AUC* area under the receiver operating characteristic curve, *CI* confidence interval, *ΔAUC* difference in AUC (FDI-C minus comparator), *DeLong* DeLong test for paired AUC comparison, *FDI-C* FEES Dysphagia Index composite, *FDI-E* FEES Dysphagia Index-Efficiency, *FDI-S* FEES Dysphagia Index-Safety, *FEES* flexible endoscopic evaluation of swallowing, *FOIS* Functional Oral Intake Scale, *HAP* hospital-acquired pneumonia (ICD-10 J69), *LOS* length of stay, *n* number, *PAS* Penetration–Aspiration Scale, *ρ* Spearman correlation coefficient

*N* = *1,257. Prolonged LOS: FDI-S ρ* =  *− 0.200 vs Worst PAS ρ* = *0.131*

Values are AUC (95% CI). Prolonged LOS in Online Resource 2

### Scale properties

FDI-C produced 387 unique values (ratio 0.249)—48 times more than Worst PAS (8 values, ratio 0.005) and nearly 100 times more than ordinal severity classifications. The number of unique FDI-C values increased with k (k = 1: 31, k = 2: 111, k = 3: 196, k = 4: 176), whereas Worst PAS naturally remained at 8 regardless of k.

For HAP, the logit relationship was linear (RCS non-linearity *p* = 0.601), and continuous treatment was preferred over quintiles by AIC (ΔAIC =  + 7.6). For FOIS ≤ 3, the relationship was borderline linear (*p* = 0.062). Calibration across deciles showed monotonic gradients for HAP (rate 37.2% → 3.8%) and FOIS ≤ 3 (90.2% → 1.6%).

FDI-C correlated strongly with ordinal FOIS (Spearman ρ = 0.731, *n* = 1,545). Mean FDI-C showed a monotonic gradient across all seven FOIS levels: 36.5 (FOIS 1, nothing by mouth), 42.4 (FOIS 2), 53.2 (FOIS 3), 54.3 (FOIS 4), 69.1 (FOIS 5, oral diet with special preparation), 74.4 (FOIS 6), and 87.2 (FOIS 7, total oral diet). In a proportional odds model, the FDI-C–FOIS relationship was perfectly linear (RCS non-linearity *p* = 0.986)—the strongest linearity result in the entire analysis.

**Fig. 2:**
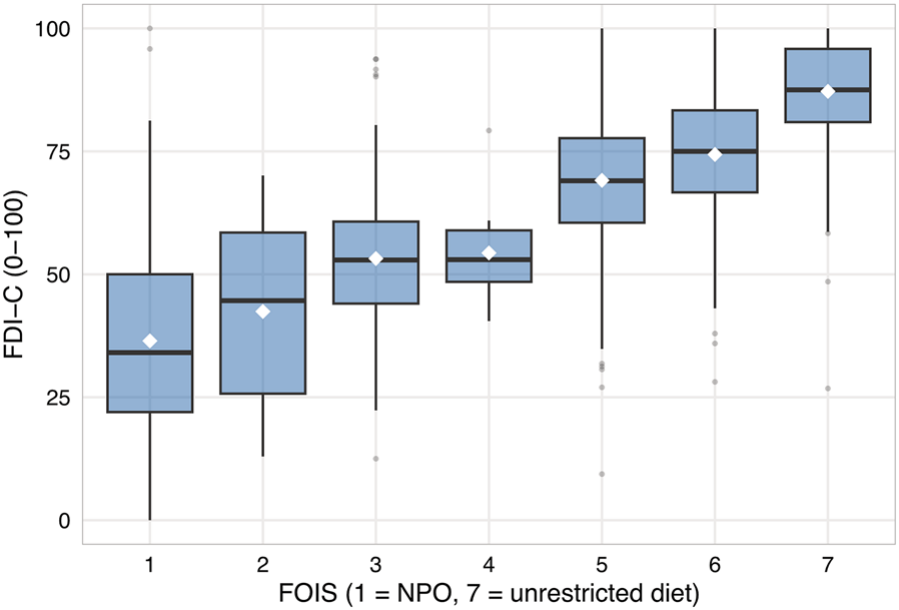
FDI-C and oral intake. Distribution of FDI-C by ordinal FOIS level (1–7) in Cohort 2. Boxes represent interquartile range, horizontal lines represent medians, whiskers extend to 1.5 times the interquartile range. Note: In this neurological inpatient cohort, FOIS = 4 (total oral intake of a single consistency) is rarely recommended; this pattern may differ in head-and-neck oncology cohorts, for which the FOIS was originally developed and single-consistency diets are more commonly prescribed. *Abbreviations:* FDI-C, FEES Dysphagia Index composite; FOIS, Functional Oral Intake Scale; Q1–Q4, FDI-C quartiles 1 (worst) to 4 (best)

Results are presented in Table 5 and Fig. 2.

**Table 5:** Scale properties of FDI-C (Cohort 2)

Property	FDI-C	Worst PAS	Ordinal FEES severity	Clinician-rated severity
Unique values	387	8	4	7
Ratio (unique/n)	0.249	0.005	0.003	0.005
RCS non-linearity p				
Asp. pneumonia	0.601 (linear)	–	–	–
Mortality	0.010 (sigmoidal)	–	–	–
FOIS ≤ 3	0.062 (borderline)	–	–	–
ΔAIC (continuous vs quintiles)				
Asp. pneumonia	+ 7.6 (continuous preferred)	–	–	–
Mortality	− 5.8 (quintiles preferred)	–	–	–
FOIS ≤ 3	+ 46.5 (continuous preferred)	–	–	–
Ordinal FOIS construct validity				
Spearman ρ vs FOIS 1–7	0.731	–	–	–
Proportional odds RCS p	0.986 (linear)	–	–	–

*AIC* Akaike information criterion, *FDI-C* FEES Dysphagia Index composite, *FEES* flexible endoscopic evaluation of swallowing, *FOIS* Functional Oral Intake Scale, *PAS* Penetration–Aspiration Scale, *RCS* restricted cubic spline (4 knots), *ρ* Spearman correlation coefficient

### FDI-C as the swallowing component of clinical decision-making

The clinical decision model confirmed that FDI-C, functional status, and diagnosis each contribute independently to predicting the clinician's FOIS recommendation. In a proportional odds model for ordinal FOIS (*n* = 1,376), all three predictors were highly significant (all *p* < 0.001). Model fit improved progressively: AIC decreased from 3,138.8 (FDI-C alone, McFadden R^2^ = 0.264) to 2,862.4 (+ SPI, R^2^ = 0.330) to 2,727.2 (+ diagnosis, R^2^ = 0.362). The full model correctly predicted the exact FOIS level in 63.1% of cases and was within ± 1 FOIS level in 77.0%.

Relative to stroke, neurodegenerative patients received more liberal diets at equivalent swallowing severity and functional status (β =  + 1.86, *p* < 0.001), while "other" diagnoses showed a smaller effect in the same direction (β =  + 0.49, *p* < 0.001). In the cross-tabulation of FDI-C quartiles by diagnosis, among patients with the worst swallowing (Q1), 78.6% of stroke patients received FOIS ≤ 3 versus only 28.9% of neurodegenerative patients. In the best quartile (Q4), the groups converged (1–3% FOIS ≤ 3).

For binary outcomes, the three-predictor model achieved AUC 0.930 for FOIS ≤ 3 (vs. 0.899 for FDI-C alone, + 0.031; each step significant at *p* < 0.001) and AUC 0.810 for HAP (vs. 0.688 for FDI-C alone, + 0.122). For HAP, the diagnosis effect was driven by neurodegenerative patients (OR = 0.242, *p* < 0.001); "other" diagnoses did not differ from stroke (OR = 0.816, *p* = 0.342).

### The mortality transition zone

**Fig. 3:**
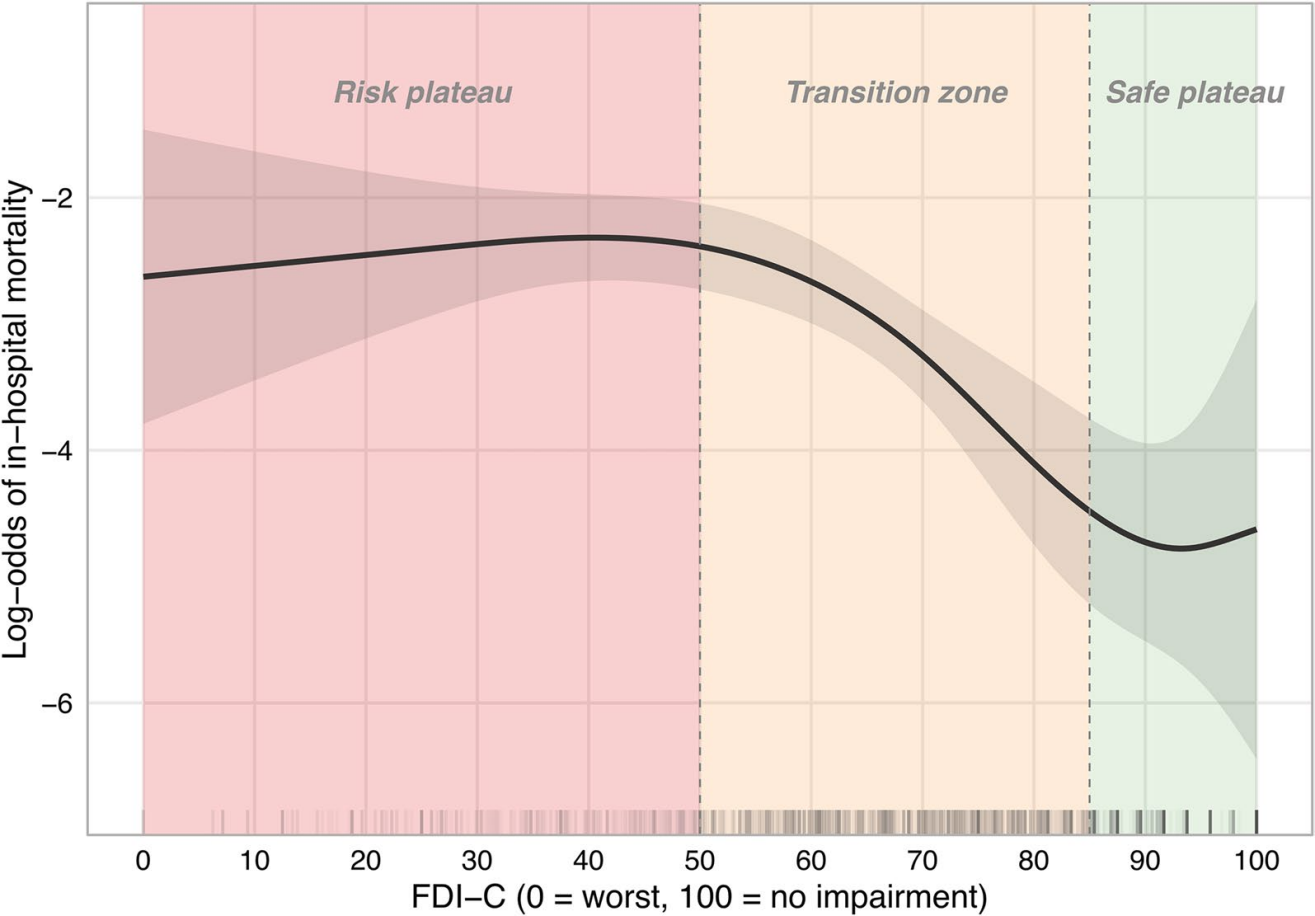
Restricted cubic spline (4 knots) showing the log odds of in-hospital mortality as a function of FDI-C (Cohort 2, *n* = 1,557, 65 deaths; non-linearity *p* = 0.010). Shaded area represents the 95% confidence band. Shaded background regions indicate approximate zone assignments: risk plateau (FDI-C < 50), transition zone (50–85), and safe plateau (> 85). Tick marks at the bottom represent individual patient FDI-C values. *Abbreviations:* FDI-C, FEES Dysphagia Index composite; n, number; RCS, restricted cubic spline

The relationship between FDI-C and in-hospital mortality did not follow the linear pattern observed for HAP (RCS non-linearity *p* = 0.010). The restricted cubic spline revealed an inverse sigmoidal curve with three approximate regions (Fig. 3), which should be interpreted as hypothesis-generating given the limited number of events (65 deaths): an approximate safe plateau above FDI-C ~ 85 (mortality 0.7–1.1% across deciles D8-D10), an approximate transition zone between FDI-C ~ 50 and ~ 85 where mortality increased steeply from approximately 1% to 7–11%, and an approximate risk plateau below FDI-C ~ 50 where mortality rates no longer increased further (D1: 7.1%, D2: 10.8%). Confidence intervals were wide, particularly in the tails (Fig. 3). 1

Results are presented in Table 6 and Fig. 3.

**Table 6:** Mortality transition zone: calibration across FDI-C deciles (Cohort 2)

Decile	n	Deaths	Mortality (%)	Mean FDI-C	Zone
D1	156	11	7.1	26.2	Risk plateau
D2	157	17	10.8	47.7	Risk plateau
D3	154	8	5.2	57.0	Transition
D4	164	13	7.9	62.7	Transition
D5	152	6	3.9	68.6	Transition
D6	152	1	0.7	73.7	Transition
D7	172	5	2.9	78.9	Transition
D8	143	1	0.7	84.4	Safe plateau
D9	177	2	1.1	90.7	Safe plateau
D10	130	1	0.8	98.8	Safe plateau

*D1–D10* FDI-C deciles 1 (lowest, worst swallowing function) to 10 (highest, best), *FDI-C* FEES Dysphagia Index composite, *n* number, *RCS* restricted cubic spline

Total: *n* = 1,557, 65 deaths (4.2%). Zone assignments approximate, based on RCS spline shape

### Inter-rater reliability

FDI introduces no rater judgment beyond PAS and Yale, unlike DIGEST-FEES, which requires frequency classification, aspiration amount estimation, and residue percentage grading [12]. We estimated composite IRR using the Spearman–Brown formula rather than direct measurement, relying on published single-consistency IRR values from other centers [8, 30, 31]. By the Spearman–Brown formula, expected κ for FDI-S (based on PAS ρ₁ = 0.85) ranges from 0.92 (k = 2) through 0.94 (k = 3) to 0.96 (k = 4), exceeding published DIGEST-FEESsafety IRR (κ_w = 0.86 [12], 0.83 [13]) from k ≥ 2 consistencies onward.

For FDI-E (based on Yale ρ₁ = 0.751), the corresponding estimates are 0.86 (k = 2), 0.90 (k = 3), and 0.92 (k = 4), confirming that the efficiency component is also a reliable measure, again exceeding published DIGEST-FEES_efficiency_ IRR (κ_w = 0.74 [12], 0.80 [13]).

The FDI-C composite reliability is thus bounded from below by the FDI-E estimates. We thus estimate the IRR of FDI-C to be κ ≥ 0.86 at a minimum, with κ ≥ 0.90 for the 84% of patients tested on three or more consistencies. This compares favorably with published DIGEST-FEES IRR (κ_w = 0.83 [12], 0.82 [13]).

### Internal validation

In the 60/40 random split, FDI-C was stable: HAP AUC derivation 0.709 vs. validation 0.687 (Δ =  − 0.022); FOIS ≤ 3 0.902 vs. 0.896 (Δ =  − 0.006); mortality 0.703 vs. 0.709 (Δ =  + 0.006). FDI-C remained significantly superior to Worst PAS in the validation set for HAP (*p* = 0.007) and FOIS ≤ 3 (*p* < 0.001), and equivalent to clinician-rated severity (all *p* ≥ 0.243). Full split AUCs for FDI-S, FDI-E, and FDI-C across all outcomes are provided in Online Resource 4.

## Discussion

### Principal findings

This study establishes the FEES Dysphagia Index through four stages: (1) identification and quantification of selection bias from clinical gating in multi-consistency FEES testing, (2) derivation of FDI-S as a bias-resilient alternative (Cohort 1, *N* = 1,257), (3) temporal replication of bias resilience and extension to efficiency (FDI-E) and a composite (FDI-C) in Cohort 2 (*N* = 1,686), and (4) demonstration that FDI-C captures the swallowing physiology component of clinical decision-making and has properties suitable for both clinical use and dysphagia research. Four findings merit detailed discussion.

### Selection bias: quantifying a known but unaddressed problem

The selection bias introduced by clinical gating has been recognized qualitatively [7–9, 11], but no study has quantified its magnitude in the context of variable consistency testing.

Our data extend these observations by providing the first formal quantification in the context of variable consistency testing. In Cohort 1, Worst PAS was inflated by 24% in complete-case analyses. In Cohort 2, the bias was 10%. The difference reflects cohort composition (Cohort 1 had higher thickened liquid testing rates), but the pattern is consistent: maximum-based scores are systematically biased by clinical gating. That FDI-S deviated by < 2% from the IPW reference in Cohort 1 and < 1% in Cohort 2 demonstrates that simple averaging inherently corrects for this bias.

The mechanism is straightforward. By dividing by the number of consistencies tested, the arithmetic mean normalizes for the variable denominator that inflates maximum statistics. The IPW analysis confirms that this correction approximates a formal causal estimand, i.e., the score that would be observed under complete testing. That a bedside-calculable arithmetic mean closely approximates the output of a formal causal inference framework without requiring any covariate information is an unexpected finding, but underscores the practical advantage of the averaging approach.

The bias resilience of FDI rests on a sequential conditional independence assumption: for each untested consistency, its potential score is independent of the testing decision, conditional on scores from previously tested consistencies and patient covariates. This is weaker than the unconditional missing-at-random (MAR) assumption because the branching-tree IPW model conditions each decision node on the strongest predictor of the gating decision: the observed PAS at the preceding step. Under a strict missing-not-at-random (MNAR) scenario, where clinicians possess additional severity information not already captured by PAS, Yale scores, demographics, frailty, and functional status, both FDI and the IPW reference could underestimate true population severity. However, three observations limit the plausible magnitude of such residual bias: (1) the propensity models showed good discrimination at each branch, (2) the Naive-IPW Delta was consistent across two independent cohorts with different testing rates and different IPW specifications, and (3) the direction of any MNAR bias is predictable (underestimation of severity), meaning that the reported Naive-IPW Delta is conservative. We therefore consider residual MNAR bias unlikely to meaningfully affect the conclusions, while acknowledging that it cannot be formally excluded.

This central finding of bias resilience has implications beyond FDI. As noted in the Introduction, all major FEES severity scores rely on maximum-based or worst-finding logic and are therefore susceptible to the same bias, while the FDI framework for all practical purposes is not. An empirical comparison between FDI and these systems would be informative, but is beyond the scope of this study.

### FDI-C captures the swallowing component of clinical decision-making

The most clinically important finding is that FDI-C, a bedside-calculable arithmetic mean, captures a substantial portion of the swallowing physiology input to the clinical diet recommendation. Three lines of evidence support this interpretation.

First, FDI-C maps linearly onto ordinal FOIS across the full clinical spectrum, with a monotonic gradient from FDI-C 36.5 (FOIS 1, nothing by mouth) to 87.2 (FOIS 7, unrestricted oral diet). In that sense, FDI-C behaves as a continuous ruler of functional swallowing capacity, with near-perfect linearity in the proportional odds model (RCS *p* = 0.986).

Second, FDI-C achieves discriminative performance equivalent to clinician-rated severity for all outcomes (all *p* ≥ 0.148), and correlates with it more strongly (ρ =  − 0.829) than the ordinal FEES severity score does (ρ = 0.611). In this regard, a simple arithmetic mean approximates the holistic assessment that experienced clinicians perform intuitively.

Third, the clinical decision model demonstrates that the expert's FOIS recommendation can be substantially reconstructed from three bedside-available variables. The full model including FDI-C, SPI and diagnosis achieves AUC 0.930 for FOIS ≤ 3, with each predictor contributing incrementally (Table 7) and indicating excellent discrimination. Crucially, the direction of the diagnosis effect validates the clinical reasoning: at equivalent swallowing severity, stroke patients (acute dysphagia, compromised immune response, recovery expected) receive more restrictive diets than neurodegenerative patients (chronic adaptation, compensatory strategies established). This can be regarded as rational clinical practice formalized in a statistical model. That the diagnosis effect was most pronounced in the worst FDI-C quartile and vanished in the best quartile further supports this interpretation: when swallowing is severely impaired, clinical context drives the diet decision; when swallowing is intact, diagnosis is irrelevant for the degree of oral intake.

**Table 7:** Clinical decision model: FDI-C + SPI + Diagnosis (Cohort 2)

Panel A: Model progression, AUC for binary outcomes		
Model	FOIS ≤ 3 (*n* = 1,376)	Asp. pneumonia (*n* = 1,386)
M1: FDI-C	0.899 (0.878–0.920)	0.688 (0.646–0.730)
M2: + SPI	0.920 (0.902–0.938)	0.799 (0.769–0.830)
M3: + Diagnosis	0.930 (0.913–0.947)	0.810 (0.782–0.839)
LR: SPI adds to M1	χ^2^ = 83.1, *p* < 0.001	χ^2^ = 119.1, *p* < 0.001
LR: Dx adds to M2	χ^2^ = 39.5, *p* < 0.001	χ^2^ = 16.6, *p* < 0.001
DeLong: M2 vs M1	+ 0.021, *p* < 0.001	+ 0.111, *p* < 0.001
DeLong: M3 vs M2	+ 0.010, *p* < 0.001	+ 0.011, *p* = 0.009

Panel B: Ordinal FOIS model (proportional odds, n = 1,376)		
Model	AIC	McFadden R^2^
M1: FDI-C	3,138.8	0.264
M2: + SPI	2,862.4	0.330
M3: + Diagnosis	2,727.2	0.362

Panel C: Full model (M3) coefficients, FOIS ≤ 3				
Predictor	β	SE	OR	*p*
FDI-C	− 0.1083	0.0071	0.897	< 0.001
SPI	− 0.0777	0.0112	0.925	< 0.001
Dx: Neurodeg. vs stroke	− 2.1594	0.4161	0.115	< 0.001
Dx: Other vs stroke	− 0.6721	0.2334	0.511	0.004

Panel D: FOIS ≤ 3 rate by FDI-C quartile and diagnosis			
FDI-C quartile	Stroke	Neurodegenerative	Other
Q1 (worst)	78.6% (*n* = 224)	28.9% (*n* = 38)	56.5% (*n* = 85)
Q2	18.7% (*n* = 203)	0.0% (*n* = 60)	10.0% (*n* = 80)
Q3	9.4% (*n* = 191)	1.2% (*n* = 86)	6.0% (*n* = 67)
Q4 (best)	2.8% (*n* = 144)	0.9% (*n* = 115)	1.2% (n = 83)

Panel E: Full model (M3) coefficients, hospital-acquired pneumonia				
Predictor	β	SE	OR	*p*
FDI-C	− 0.0189	0.0040	0.981	< 0.001
SPI	− 0.0994	0.0114	0.905	< 0.001
Dx: Neurodeg. vs stroke	− 1.4182	0.4097	0.242	< 0.001
Dx: Other vs stroke	− 0.2028	0.2135	0.816	0.342

*AIC* Akaike information criterion, *AUC* area under the receiver operating characteristic curve, *β* regression coefficient, *χ*^*2*^ Chi-squared statistic, *CI* confidence interval, *DeLong* DeLong test for paired AUC comparison, *FDI-C* FEES Dysphagia Index composite, *FOIS* Functional Oral Intake Scale, *HAP* hospital-acquired pneumonia (ICD-10 J69), *LR* likelihood ratio test, *McFadden R*^*2*^ McFadden pseudo-R^2^, *n* number, *OR* odds ratio, *Q1–Q4* FDI-C quartiles 1 (worst) to 4 (best), *SE* standard error, *SPI* Self-Care Index

Prediction accuracy (M3): exact FOIS match 63.1%, within ± 1 level 77.0%

The implication is that FDI-C, together with functional status and diagnostic context, can approximate the FOIS level that an experienced clinician would recommend. This has the potential to standardize dysphagia severity assessment across settings, pending external validation.

### The mortality transition zone

The sigmoidal FDI-C–mortality relationship described above is more clinically informative than a linear gradient: If confirmed, the safe plateau would suggest that mild dysphagia does not contribute meaningfully to mortality risk. The apparent transition zone may identify a range where increasing swallowing impairment begins to matter: aspiration risk accumulates, nutritional status deteriorates, and clinical interventions become necessary. The apparent saturation below FDI-C ~ 50 may reflect an intervention ceiling: patients with severe dysphagia are placed on maximal precautions (nil per os, tube feeding), so that further deterioration in swallowing physiology no longer translates into additional mortality.

With only 65 deaths and wide confidence intervals, particularly in the tails, this pattern should be considered hypothesis-generating. If confirmed in larger cohorts with sufficient events to test whether the threshold shifts by diagnosis or acuity, the transition zone could represent a suitable target range for clinical intervention and a natural enrichment criterion for therapeutic trials.

### FDI-C as a dual-use instrument

#### FDI-C has properties that position it for two complementary roles

*Clinical decision support.* The linear FDI-C–FOIS mapping and the clinical decision model (AUC 0.930 for FOIS ≤ 3) suggest that FDI-C could standardize how FEES findings are translated into diet recommendations. Unlike existing ordinal scores, which require subjective severity grading, FDI-C is computed directly from PAS and Yale ratings, i.e., the same standardized scales that FEES examiners already document. By making the link between swallowing physiology and diet recommendation explicit and reproducible, FDI-C may reduce inter-examiner variability in FEES interpretation—a known problem across centers [10]. Pending prospective validation, a conversion chart between FDI-C ranges and recommended FOIS levels (or ranges), stratified by stroke/non-stroke cohorts could serve as a practical decision aid. Also, the FDI would facilitate tracking the recovery trajectories of patients across centers (acute care–rehabilitation facilities–outpatient clinics) in the same way.

*Research endpoint.* FDI-C produces 387 unique values with confirmed linearity for the primary outcome (HAP: RCS p = 0.60). By the asymptotic relative efficiency framework [26], categorizing a continuous variable into five ordinal levels requires approximately 10–15% more subjects. When the superior discrimination of FDI-C (which will further reduce unexplained variance in trial data) is additionally considered, the combined effect translates to approximately 15–22% fewer subjects required to detect a given effect size. FEDSS has been used as the primary endpoint in at least two published RCTs [34, 35]; FDI could serve the same role with greater statistical efficiency.

The equal weighting of FDI-S and FDI-E deserves comment. Regression-derived optimal weights for FDI-S ranged from 0.51 (mortality) to 0.77 (FOIS), reflecting outcome-specific differences in the relative importance of safety versus efficiency. However, the AUC differences between optimally weighted and equally weighted composites were negligible for the distally measured outcomes and no DeLong comparison reached significance (all p > 0.5). Bootstrap 95% confidence intervals for the AUC-maximizing weight included 0.50 for three of four outcomes. The exception was FOIS, where the aspiration-dominated PAS component naturally dominates; however, this is the outcome most affected by the circularity limitation noted above and therefore least suitable for driving weight optimization. Equal weighting thus represents a principled, outcome-agnostic default that avoids overfitting (Online Resource 3).

That a single score can serve both roles simultaneously—guiding clinical decisions at the bedside while providing a continuous, bias-resilient outcome measure for research—is unusual in dysphagia assessment. Existing scores tend to be optimized for one purpose or the other. FDI-C bridges this gap by being simple enough for clinical use and precise enough for parametric analysis. It is also fully compatible with the integrated FEES report proposed by Dziewas and colleagues [4], which structures the clinical workflow from salient findings through severity grading and phenotyping to individualized treatment recommendations. FDI-C could serve as the central quantitative severity metric within this framework (Step 3), replacing the ordinal FEES dysphagia score with a continuous, bias-resilient alternative while preserving the framework's clinical logic.

### Limitations

First, this is a single-center study. While the temporal replication across two cohorts separated by a multi-year gap provides stronger evidence than a single-cohort analysis, both cohorts share the same protocol, clinical team, and patient population. The FDI-C–FOIS mapping in particular may reflect institutional practice patterns. External validation in neurological and non-neurological cohorts is a logical and necessary next step; collaborations with external centers are ongoing.

Cross-center applicability also depends on consistency classification systems. The International Dysphagia Diet Standardisation Initiative (IDDSI) framework is increasingly adopted internationally [36]. FDI computation is agnostic to the specific consistency taxonomy (it averages across whatever consistencies are tested) but the specific FDI-C–FOIS mapping may vary with institutional consistency protocols and IDDSI levels used.

Second, the Cohort 2 IPW estimates for the thickened liquid branch were unstable (positivity violation at 35% testing rate, weights up to 39.3). However, the sensitivity analysis confirmed that the Naive-IPW Delta remained < 1%, and Cohort 1's well-behaved IPW (range 0.20–5.45) provides independent confirmation.

Third, the FOIS outcome is subject to some circularity: the FEES examiner is typically the same clinician who recommends FOIS. This circularity is inherent to the clinical workflow: FDI-C captures the swallowing information that informs the FOIS decision, so high correspondence is expected by design. Still, the distally measured outcomes (HAP, mortality) provide independent validation unaffected by this circularity.

Fourth, we operationalized our primary respiratory outcome as hospital-acquired pneumonia (HAP) using ICD-10 J69 coding during the inpatient stay. This is a pragmatic but imperfect proxy: J69 captures aspiration pneumonitis, bacterial pneumonia of presumed aspiration origin, and chemical pneumonitis, and retrospective coding cannot distinguish these entities; coding accuracy is also known to be variable across institutions. We therefore report HAP rather than "aspiration pneumonia" to avoid overstating the etiological attribution.

Fifth, FDI inherits PAS and Yale psychometric limitations. However, by averaging across consistencies, FDI mathematically reduces measurement error (Spearman-Brown effect), yielding expected composite reliability exceeding DIGEST-FEES from k ≥ 2 onward.

Sixth, the mortality transition zone is based on just 65 events and therefore should be considered hypothesis-generating only. Confirmation in larger cohorts, ideally with sufficient events to test whether the threshold shifts by neurological diagnosis, is needed.

Seventh, we present no responsiveness-to-change data yet. The continuous nature of FDI-C suggests sensitivity to longitudinal change, but this requires demonstration.

Eighth, FDI operates on the clinician-documented PAS per consistency, typically recorded as the worst PAS across three to four swallow trials of that consistency [37]. By the same order-statistics logic that motivates the between-consistency averaging in FDI, this within-consistency maximum convention inflates per-consistency scores relative to a trial-level mean. Because the convention is uniform across all PAS-based FEES scores, it does not differentially disadvantage FDI; however, a trial-level FDI variant averaging all documented swallow trials rather than consistency summaries would be even more bias-resilient and is a logical extension pending routine trial-level documentation.

Ninth, although the FDI framework is conceptually agnostic to the imaging modality and PAS and Yale scores are equally applicable to videofluoroscopic swallow studies (VFSS, also termed Modified Barium Swallow study), we developed and validated FDI exclusively in FEES cohorts. VFSS differs in consistency protocols, trial structure, and rating conditions, and is typically scored with the Modified Barium Swallow Impairment Profile (MBSImp, [38]) rather than with consistency-summarized PAS/Yale. A parallel FEES/VFSS validation cohort, in which the same patients are examined with both modalities and FDI is computed alongside MBSImp component and overall scores, would directly address whether FDI's bias resilience and predictive validity generalize across imaging modalities; such head-to-head comparisons are a natural next step in the broader program of FDI external validation.

## Conclusions

The FEES Dysphagia Index, combining swallowing safety (PAS) and efficiency (Yale residue) via simple averaging, is a bias-resilient, bedside-calculable continuous score that captures the swallowing physiology component of expert clinical judgment. Derived in 1,257 patients and temporally replicated in 1,686, it produces 387 unique values with confirmed interval-scale properties and maps linearly onto clinician-assigned oral intake levels. Combined with functional status and diagnostic context, FDI-C reconstructs the expert's diet recommendation with high accuracy and reliably predicts HAP with AUC 0.81. The sigmoidal relationship with mortality suggests a clinically meaningful transition zone that may inform enrichment strategies for therapeutic trials. These properties position FDI-C as both a clinical decision support tool and a continuous endpoint for dysphagia research, pending external validation.

## Supplementary Information

Below is the link to the electronic supplementary material.

Supplementary file1 (DOCX 40 kb)

## Data Availability

The dataset is not publicly available due to patient privacy regulations. Anonymized summary data and analysis code are available from the corresponding author upon reasonable request.
