# Supplementary material for: The FEES Dysphagia Index: a bias-resilient continuous score that captures expert clinical judgment in 2,943 neurological inpatients
Source: J Neurol. 2026 Jun 3;273(6):359. doi: 10.1007/s00415-026-13895-x (PMC13234040; doi:10.1007/s00415-026-13895-x)
Supplement: Supplementary file 1 — Supplementary file1 (DOCX 40 kb) [file 415_2026_13895_MOESM1_ESM.docx]

# Online Resources

## Werner CJ et al. — The FEES Dysphagia Index: a bias-resilient continuous score that captures expert clinical judgment in 2,943 neurological inpatients

*Journal of Neurology*

## Online Resource 1 — IPW diagnostics

Branch-specific propensity-model details, diagnostics, and the full prose specification of the sequential branching-tree IPW.

### Sequential branching-tree specification

At each decision node (puree→liquid, liquid→solid, liquid→thickened liquid), a logistic propensity model estimated the probability of proceeding to the next consistency. Baseline covariates included age, sex, Hospital Frailty Risk Score (HFRS) [1], functional status (Self-Care Index at admission, SPI [2, 3]), and neurological diagnosis. PAS scores from preceding consistencies were included at all branches.

In Cohort 2, the IPW specification was refined based on Cohort 1 experience: Yale residue scores were included as additional covariates only where likelihood ratio tests confirmed their predictive relevance (liquid→solid: p < 0.001), and excluded from the liquid→thickened liquid branch a priori, as this "rescue" branch reflects a pure safety decision. Propensity scores were truncated at the 1st and 99th percentiles.

### Stabilized-weight summaries

**Cohort 1.** Stabilized weights had mean 1.00 (SD 0.87), median 0.61, range 0.20–5.45. No extreme-weight trimming was required.

**Cohort 2.** Weights for the puree→liquid and liquid→solid branches were well-behaved (max 5.48). The thickened-liquid branch showed unstable weights (max 39.3, 175 weights > 10) due to a positivity violation at a relatively small 35% testing rate. A sensitivity analysis excluding this branch was performed (see Online Resource 6).

### Cohort 1 — branch detail

Three branches (puree → thin liquid, thin liquid → solid, thin liquid → thickened liquid). Baseline covariates at all branches: age, sex, HFRS, SPI, diagnosis. PAS scores from preceding consistencies included at all branches.

| Branch | n | Propensity range | Weight range | Max weight | Weights > 10 |
| --- | --- | --- | --- | --- | --- |
| Puree → Thin liquid | 1,145 | 0.72–0.99 | 0.20–2.83 | 2.83 | 0 |
| Thin liquid → Solid | 1,062 | 0.42–0.96 | 0.37–5.45 | 5.45 | 0 |
| Thin liquid → Thickened liquid | 1,062 | 0.18–0.78 | 0.43–4.12 | 4.12 | 0 |

Stabilized weight summary: mean 1.00 (SD 0.87), median 0.61, range 0.20–5.45.

### Cohort 2 — branch detail

Three branches (puree → liquid, liquid → solid, liquid → thickened liquid). Baseline covariates: age, sex, HFRS, SPI, diagnosis. PAS from preceding consistencies at all branches. Yale residue scores included only where likelihood ratio (LR) tests confirmed predictive relevance.

| Branch | n | Yale included? | LR test p | Propensity range | Weight range | Max weight | Weights > 10 |
| --- | --- | --- | --- | --- | --- | --- | --- |
| Puree → Liquid | 1,539 | No | 0.231 | 0.68–0.99 | 0.22–3.10 | 3.10 | 0 |
| Liquid → Solid | 1,480 | Yes | < 0.001 | 0.40–0.97 | 0.35–5.48 | 5.48 | 0 |
| Liquid → Thickened liquid | 1,480 | No (a priori) | – | 0.08–0.72 | 0.47–39.3 | 39.3 | 175 |

The thickened liquid branch showed a positivity violation at a 35% testing rate, resulting in unstable weights. Propensity scores were truncated at the 1st and 99th percentiles.

## Online Resource 2 — Prolonged LOS results (Cohort 2)

Prolonged LOS defined as > median (16 days), prevalence 49.6%.

| Score | Prolonged LOS AUC (95% CI) |
| --- | --- |
| FDI-C | 0.614 (0.586–0.641) |
| FDI-S | 0.603 (0.575–0.631) |
| FDI-E | 0.596 (0.568–0.624) |
| Worst PAS | 0.559 (0.531–0.587) |
| Ordinal FEES severity score | 0.582 (0.556–0.607) |
| Clinician-rated severity | 0.629 (0.604–0.655) |
| FDI-C + SPI (clinical model) | 0.701 (0.673–0.728) |

DeLong comparisons: FDI-C vs Worst PAS: ΔAUC = +0.055, p = 0.006. FDI-C vs Clinician-rated severity: ΔAUC = −0.016, p = 0.419. RCS non-linearity p = 0.006.

## Online Resource 3 — FDI-S/FDI-E weighting sensitivity analysis

Sensitivity analysis examining whether alternative weightings of FDI-S (Safety) and FDI-E (Efficiency) improve the predictive performance of FDI-C over the equal-weight specification (FDI-C = 0.5 × FDI-S + 0.5 × FDI-E). Analyses performed in Cohort 2 (N = 1,557 with both FDI-S and FDI-E available).

### Panel A: Regression-derived optimal weights

For each outcome, a logistic regression model was fitted with standardized FDI-S and FDI-E as separate predictors. The regression-derived weight for FDI-S was computed as w_S = |β*S| / (|β*S| + |β_E|).

| Outcome | β_S (std) | β_E (std) | Ratio | w_S (regr) | w_E (regr) |
| --- | --- | --- | --- | --- | --- |
| HAP (J69) | −0.499 | −0.268 | 1.86 | 0.651 | 0.349 |
| Mortality | −0.338 | −0.328 | 1.03 | 0.508 | 0.492 |
| Prolonged LOS | −0.291 | −0.214 | 1.36 | 0.577 | 0.423 |
| FOIS ≤ 3 | −1.987 | −0.597 | 3.33 | 0.769 | 0.231 |
| **Mean** |  |  |  | **0.626** | **0.374** |

Ratio = |β*S| / |β*E|.

All β coefficients from logistic regression with standardized predictors (both p < 0.05 for all outcomes).

### Panel B: AUC across weight grid

FDI-C(w) = w × FDI-S + (1 − w) × FDI-E, with w from 0.00 to 1.00 (step 0.10).

| w_S | HAP | Mortality | LOS | FOIS ≤ 3 |
| --- | --- | --- | --- | --- |
| 0.00 | 0.656 | 0.672 | 0.596 | 0.777 |
| 0.10 | 0.669 | 0.685 | 0.602 | 0.809 |
| 0.20 | 0.680 | 0.693 | 0.606 | 0.839 |
| 0.30 | 0.688 | 0.700 | 0.610 | 0.864 |
| 0.40 | 0.695 | 0.705 | 0.612 | 0.885 |
| **0.50*** | **0.698** | **0.706** | **0.614** | **0.900** |
| 0.60 | 0.699 | 0.706 | 0.614 | 0.908 |
| 0.70 | 0.699 | 0.704 | 0.613 | 0.910 |
| 0.80 | 0.697 | 0.702 | 0.613 | 0.909 |
| 0.90 | 0.695 | 0.699 | 0.611 | 0.905 |
| 1.00 | 0.691 | 0.693 | 0.603 | 0.899 |

* Equal weight (manuscript default). AUC surfaces are flat near the optimum for HAP, mortality, and LOS, indicating insensitivity to the weighting choice.

### Panel C: AUC-maximizing weights, DeLong comparisons, and bootstrap CIs

| Outcome | Best w_S | AUC (best) | AUC (0.50) | ΔAUC | DeLong p | Bootstrap 95% CI |
| --- | --- | --- | --- | --- | --- | --- |
| HAP (J69) | 0.65 | 0.699 | 0.698 | +0.001 | 0.797 | [0.40, 1.00] |
| Mortality | 0.50 | 0.706 | 0.706 | 0.000 | 0.507 | [0.15, 0.95] |
| Prolonged LOS | 0.55 | 0.614 | 0.614 | +0.000 | 0.955 | [0.25, 0.95] |
| FOIS ≤ 3 | 0.75 | 0.910 | 0.900 | +0.010 | 0.007 | [0.65, 0.80] |

DeLong tests compare AUC-maximizing weighted FDI-C (from Panel B grid search) against equal-weighted FDI-C. Bootstrap 95% CIs based on 1,000 resamples. The bootstrap CI includes 0.50 for three of four outcomes. The DeLong test for FOIS is significant (p = 0.007), but FOIS is the outcome most affected by the circularity limitation (see main text). FDI-S and FDI-E correlation: Pearson r = 0.538.

### Interpretation

Regression-derived optimal weights are outcome-specific (w_S range: 0.51–0.77), confirming that no single alternative weighting uniformly outperforms equal weighting. For the distally measured, clinically meaningful outcomes (aspiration pneumonia, mortality), AUC differences between optimal and equal weighting are negligible (ΔAUC ≤ 0.001) and statistically non-significant. The equal-weight specification is justified as an outcome-agnostic, overfitting-resistant default that maintains clinical interpretability.

## Online Resource 4 — Derivation/Validation split AUCs (Cohort 2)

Stratified 60/40 random split (derivation n = 1,011, validation n = 675). Stability classification: |ΔAUC| < 0.02 = **stable**, 0.02–0.05 = **moderate**, > 0.05 = **unstable**.

| Score | Outcome | Derivation AUC | Validation AUC | ΔAUC | Stability |
| --- | --- | --- | --- | --- | --- |
| FDI-C | Aspiration pneumonia | 0.709 | 0.687 | −0.022 | Moderate |
| FDI-C | Mortality | 0.703 | 0.709 | +0.006 | Stable |
| FDI-C | FOIS ≤ 3 | 0.902 | 0.896 | −0.006 | Stable |
| FDI-S | Aspiration pneumonia | 0.700 | 0.675 | −0.025 | Moderate |
| FDI-S | Mortality | 0.696 | 0.693 | −0.003 | Stable |
| FDI-S | FOIS ≤ 3 | 0.901 | 0.890 | −0.011 | Stable |
| FDI-E | Aspiration pneumonia | 0.660 | 0.653 | −0.007 | Stable |
| FDI-E | Mortality | 0.654 | 0.697 | +0.043 | Moderate |
| FDI-E | FOIS ≤ 3 | 0.790 | 0.757 | −0.033 | Moderate |

FDI-C remained significantly superior to Worst PAS in the validation set for aspiration pneumonia (p = 0.007) and FOIS ≤ 3 (p < 0.001), and equivalent to clinician-rated severity (all p ≥ 0.243).

## Online Resource 5 — Inter-consistency correlations

### Cohort 1 (PAS, Spearman)

| Pair | ρ | p | n |
| --- | --- | --- | --- |
| Puree vs Thin liquid | 0.329 | < 0.001 | 1,062 |
| Puree vs Thickened liquid | 0.316 | < 0.001 | 521 |
| Puree vs Solid | 0.209 | < 0.001 | 741 |
| Thin liquid vs Thickened liquid | 0.320 | < 0.001 | 532 |
| Thin liquid vs Solid | 0.135 | < 0.001 | 763 |
| Thickened liquid vs Solid | 0.183 | 0.003 | 350 |

### Cohort 2 (PAS, Spearman)

| Pair | ρ | p | n |
| --- | --- | --- | --- |
| Puree vs Liquid | 0.325 | < 0.001 | 1,480 |
| Puree vs Thickened liquid | 0.243 | < 0.001 | 592 |
| Puree vs Solid | 0.141 | < 0.001 | 1,136 |
| Liquid vs Thickened liquid | 0.150 | < 0.001 | 589 |
| Liquid vs Solid | 0.130 | < 0.001 | 1,142 |
| Thickened liquid vs Solid | 0.151 | < 0.001 | 417 |

### Cohort 2 (Yale combined, Spearman)

| Pair | ρ | p | n |
| --- | --- | --- | --- |
| Puree vs Liquid | 0.527 | < 0.001 | 1,472 |
| Puree vs Thickened liquid | 0.472 | < 0.001 | 589 |
| Puree vs Solid | 0.539 | < 0.001 | 1,145 |
| Liquid vs Thickened liquid | 0.599 | < 0.001 | 583 |
| Liquid vs Solid | 0.377 | < 0.001 | 1,141 |
| Thickened liquid vs Solid | 0.446 | < 0.001 | 418 |

PAS correlations are moderate (ρ = 0.13–0.33), confirming that swallowing safety with one consistency does not reliably predict safety with others. Yale correlations are higher (ρ = 0.38–0.60), consistent with pharyngeal residue being a more stable trait.

## Online Resource 6 — Sensitivity analysis: IPW with vs. without thickened liquid branch (Cohort 2)

### Three-cohort bias comparison under both IPW specifications

| Score | Subcohort | IPW full model | IPW no-thick model |
| --- | --- | --- | --- |
| FDI-S | CC mean (n = 412) | 66.97 | 66.97 |
|  | SCD mean (n = 1,480) | 67.24 | 67.24 |
|  | IPW reference | 65.70 | 74.13 |
|  | Naive-IPW Delta | 0.5% | 0.4% |
| FDI-E | CC mean (n = 412) | 61.45 | 61.45 |
|  | SCD mean (n = 1,480) | 65.04 | 65.04 |
|  | IPW reference | 61.90 | 65.58 |
|  | Naive-IPW Delta | 0.6% | 0.3% |
| FDI-C | CC mean (n = 412) | 64.21 | 64.21 |
|  | SCD mean (n = 1,480) | 66.14 | 66.14 |
|  | IPW reference | 63.81 | 69.73 |
|  | Naive-IPW Delta | 0.6% | 0.4% |
| Worst PAS | CC mean (n = 412) | 7.02 | 7.02 |
|  | SCD mean (n = 1,480) | 4.84 | 4.84 |
|  | IPW reference | 6.39 | 4.71 |

The IPW reference values diverge substantially between the two specifications (e.g., FDI-S: 65.70 vs 74.13, Δ = 8.43 points), confirming that the exact IPW-corrected population mean is sensitive to modeling choices in the presence of positivity violations. However, the Naive-IPW Delta remains below 1% under both specifications for all FDI variants, demonstrating that FDI's bias resilience is not dependent on any particular IPW model.

## Online Resource 7 — Yale weighting sensitivity analysis (Cohort 2)

### Panel A: Pooled Yale-FOIS regression (cluster-robust, N = 4,755 observations from 1,555 patients)

| Predictor | β | p | Relative weight |
| --- | --- | --- | --- |
| Yale Valleculae | −0.211 | < 0.001 | 0.29 |
| Yale Sinus Piriformis | −0.527 | < 0.001 | 0.71 |

R² = 0.184. Cluster-robust standard errors (patient-level clustering).

### Panel B: Per-consistency Yale-FOIS regressions

| Consistency | n | R² | β Vall (p) | β SP (p) | Weight Vall : SP |
| --- | --- | --- | --- | --- | --- |
| Puree | 1,467 | 0.179 | −0.139 (< 0.001) | −0.579 (< 0.001) | 0.19 : 0.81 |
| Liquid | 1,445 | 0.082 | −0.254 (0.002) | −0.435 (< 0.001) | 0.37 : 0.63 |
| Thickened liquid | 591 | 0.056 | −0.246 (0.002) | −0.101 (0.136) | 0.71 : 0.29 |
| Solid | 1,143 | 0.125 | −0.251 (< 0.001) | −0.198 (< 0.001) | 0.56 : 0.44 |

Relative weights vary across consistencies and reverse between thickened liquid (valleculae-dominant) and puree (pyriform sinus-dominant).

### Panel C: FOIS correlation comparison (simple mean vs. empirically weighted Yale)

| Consistency | ρ (simple mean, FOIS) | ρ (weighted, FOIS) | Δρ |
| --- | --- | --- | --- |
| Puree | −0.495 | −0.478 | +0.017 |
| Liquid | −0.358 | −0.356 | +0.002 |
| Thickened liquid | −0.208 | −0.205 | +0.004 |
| Solid | −0.387 | −0.377 | +0.011 |

The simple mean achieves equal or marginally higher FOIS correlations across all consistencies.

### Panel D: FDI-E variant comparison

| Variant | Mean (SD) | Pearson r |
| --- | --- | --- |
| FDI-E (simple mean) | 64.5 (21.3) | – |
| FDI-E (weighted, 0.29 × Vall + 0.71 × SP) | 67.8 (21.6) | 0.9845 |

The two FDI-E variants are near-identical (r = 0.98). Because the relative weight of valleculae vs. pyriform sinuses reverses between consistencies (Panel B), the pooled empirical weights do not generalize across testing protocols. The simple mean was retained for parsimony and generalizability.

## Online Resource 8 — STROBE checklist for cohort studies

Checklist of items that should be included in reports of cohort studies, per von Elm et al. (2007), *Lancet* 370:1453–1457.

| # | Item | Recommendation | Reported in |
| --- | --- | --- | --- |
| **Title and abstract** |  |  |  |
| 1 | (a) Indicate the study's design with a commonly used term in the title or the abstract | "retrospective two-cohort study" | p. 1 (Title); p. 2 (Abstract) |
| 2 | (b) Provide in the abstract an informative and balanced summary of what was done and what was found | Study design, methods, key results, and conclusion summarized | p. 2 (Abstract) |
| **Introduction** |  |  |  |
| 3 | Explain the scientific background and rationale for the investigation being reported | Selection bias from clinical gating; limitations of existing scores | pp. 3–4 (Introduction) |
| 4 | State specific objectives, including any prespecified hypotheses | Four aims stated | p. 4 (Introduction) |
| **Methods** |  |  |  |
| 5 | Present key elements of study design early in the paper | Retrospective two-cohort, derivation + temporal replication | p. 5 (Methods → Study design) |
| 6 | Describe the setting, locations, and relevant dates | Single center (RWTH Aachen); Cohort 1: 2013–2018; Cohort 2: 2021–2025 | p. 5 (Methods → Study design) |
|  | (a) Give the eligibility criteria, and the sources and methods of selection of participants | Consecutive neurological inpatients undergoing FEES | p. 5 (Methods → Study design) |
|  | (b) For matched studies — give matching criteria and number of exposed and unexposed | N/A (not a matched study) | — |
| 7 | Clearly define all outcomes, exposures, predictors, potential confounders, and effect modifiers | FDI-S, FDI-E, FDI-C defined; outcomes (HAP, mortality, FOIS, LOS) defined; IPW covariates specified | pp. 5–6 (Methods → FDI construction, Comparator scores, Clinical outcomes); Online Resource 1 |
| 8 | For each variable of interest, give sources of data and details of methods of assessment | PAS [REF #6], Yale [REF #20], FOIS [REF #21], ICD-10 J69, HFRS [REF #23], SPI [REF #24], [REF #25] | pp. 5–6 (Methods → FEES protocol, Clinical outcomes) |
| 9 | Describe any efforts to address potential sources of bias | Sequential branching-tree IPW; three-cohort bias design; sensitivity analysis (thick branch); temporal replication | pp. 6–7 (Methods → Inverse probability weighting, Bias quantification); Online Resources 1, 6, 9 |
| 10 | Explain how the study size was arrived at | Consecutive patients in defined time windows; no a priori sample size calculation (retrospective) | p. 5 (Methods → Study design) |
| 11 | Explain how quantitative variables were handled in the analyses | FDI continuous (0–100); RCS for non-linearity; binary outcomes defined | pp. 5–7 (Methods → FDI construction, Scale properties, Predictive validity); Online Resource 10 |
| 12 | (a) Describe all statistical methods, including those used to control for confounding | IPW, DeLong AUC comparisons, logistic regression, proportional odds, RCS, Spearman-Brown | pp. 6–7 (Methods → Inverse probability weighting, Predictive validity, Scale properties, Clinical decision model, Inter-rater reliability, Statistical analysis); Online Resources 1, 9, 10, 11, 12 |
|  | (b) Describe any methods used to examine subgroups and interactions | Diagnosis-stratified decision model; FDI-C quartile × diagnosis cross-tabulation | p. 8 (Methods → Clinical decision model); Results (Cohort characteristics) |
|  | (c) Explain how missing data were addressed | FOIS missing (n = 63) treated as missing with chart-review characterization; IPW for consistency-level missingness | Results → Cohort characteristics; pp. 6–7 (Methods → Inverse probability weighting); Online Resource 9 |
|  | (d) If applicable, describe analytical methods taking account of sampling strategy | Stratified 60/40 derivation/validation split | p. 8 (Methods → Internal validation); Online Resource 4 |
|  | (e) Describe any sensitivity analyses | IPW without thick branch; FDI-S/FDI-E weighting; Yale weighting | p. 6 (Methods → FDI construction, Bias quantification); Online Resources 3, 6, 7 |
| **Results** |  |  |  |
| 13 | (a) Report numbers of individuals at each stage of study | N = 2,943 total; Cohort 1 N = 1,257; Cohort 2 N = 1,686; complete cases n = 412 | p. 10 (Results → Cohort characteristics, Consistency testing patterns) |
|  | (b) Give reasons for non-participation at each stage | 63 FOIS-missing characterized (failed exams, documentation gaps, not recorded) | p. 10 (Results → Cohort characteristics) |
|  | (c) Consider use of a flow diagram | Not included (consecutive cohort, no exclusions beyond FEES status) | — |
| 14 | (a) Give characteristics of study participants and information on exposures and potential confounders | Age, sex, diagnosis, HFRS, SPI, outcome rates | p. 10-11 (Results → Cohort characteristics); Table 1 |
|  | (b) Indicate number of participants with missing data for each variable of interest | Testing rates per consistency; FOIS missing n = 63 | p. 10 (Results → Consistency testing patterns, Cohort characteristics) |
| 15 | Report numbers of outcome events or summary measures over time | HAP, mortality, FOIS ≤ 3, prolonged LOS counts and prevalences | p. 10-11 (Results → Cohort characteristics); Table 1 |
| 16 | (a) Give unadjusted estimates and, if applicable, confounder-adjusted estimates and their precision | AUCs with 95% CIs (Table 4); IPW-adjusted vs. naive AUCs | pp. 11–12 (Results → Bias resilience, Predictive validity); Table 4 |
|  | (b) Report category boundaries when continuous variables were categorized | FDI-C deciles for calibration; FOIS ≤ 3 threshold; LOS > median | p. 13 (Results → Scale properties); p. 6 (Methods → Clinical outcomes) |
|  | (c) If relevant, consider translating estimates of relative risk into absolute risk for a meaningful time period | Mortality rates per FDI-C decile (Table 7) | p. 15 (Results → Mortality transition zone); Table 7 |
| 17 | Report other analyses done | Derivation/validation split; IPW sensitivity; weighting sensitivity; diagnosis × FDI-C interaction | p. 17 (Results → Internal validation); pp. 9, 19; Online Resources 3, 4, 6 |
| **Discussion** |  |  |  |
| 18 | Summarise key results with reference to study objectives | Four-stage summary | p. 18 (Discussion → Principal findings) |
| 19 | Discuss limitations of the study, taking into account sources of potential bias or imprecision | Single-center; IPW instability; FOIS circularity; J69 coding; IRR estimated; mortality events; no responsiveness data; within-consistency sampling; FEES-only validation (VFSS/MBSImp future direction) | pp. 21-23 (Discussion → Limitations; nine items) |
| 20 | Give a cautious overall interpretation of results | Findings contextualized; mortality zone explicitly hypothesis-generating; external validation noted as necessary | pp. 18–23 (Discussion throughout) |
| 21 | Discuss the generalisability (external validity) of the study results | Single-center limitation acknowledged; IDDSI compatibility discussed; external collaborations mentioned; VFSS/MBSImp comparison flagged as next step | p. 21 (Discussion → Limitations) |
| **Other information** |  |  |  |
| 22 | Give the source of funding and the role of the funders | No external funding | p. 28 (Statements and Declarations) |

Reference: von Elm E, Altman DG, Egger M, Pocock SJ, Gøtzsche PC, Vandenbroucke JP. The Strengthening the Reporting of Observational Studies in Epidemiology (STROBE) statement: guidelines for reporting observational studies. *Lancet* 2007;370:1453–1457.

## Online Resource 9 — Bias-quantification assumptions

Both the IPW framework and the FDI averaging approach assume **sequential conditional independence** (sequential missing-at-random / MAR): at each testing decision, the missingness of untested consistencies is independent of their potential outcomes, conditional on observed covariates including PAS and Yale scores from previously tested consistencies. Because the branching-tree model conditions each decision node on the strongest clinical predictor of the subsequent gating decision — namely the swallowing outcome observed at the preceding step — this assumption is substantially weaker than unconditional MAR.

### Direction of any residual bias under MNAR

If clinical gating is informative beyond what the observed covariates capture (a missing-not-at-random / MNAR scenario), the most plausible mechanism is that clinicians withhold a consistency precisely when they expect a worse outcome at that consistency. Under this scenario:

- The IPW reference value slightly **underestimates** true population severity (because patients with the worst expected scores at untested consistencies are reweighted using observed scores from a "safer" tested distribution).
- The naive FDI average is computed only over tested consistencies, so it is sensitive to the same mechanism but does not invoke any modeling assumption.
- The **Naive-IPW Delta** therefore represents a conservative estimate of FDI's bias resilience: both estimators move in the same direction under MNAR, so their difference under-states the true MNAR-induced bias in either one alone.

In practical terms, the observed Naive-IPW Delta < 1% across all FDI variants and across both IPW specifications (full and thick-excluded; Online Resource 6) indicates that any residual MNAR-induced bias is small relative to the inter-individual variability in FDI and well within clinically meaningful precision.

### Empirical check

The cross-IPW-specification consistency reported in Online Resource 6 — naive mean unchanged, IPW reference shifting by up to 8.4 points, Naive-IPW Delta < 1% under both specifications — provides empirical evidence that FDI's bias resilience does not rest on any particular modeling assumption. The MAR/IPW framework is used to *quantify* bias; the FDI averaging principle is what *delivers* bias resilience, by construction.

## Online Resource 10 — Scale-properties methodology (Cohort 2)

We assessed whether FDI-C meets the empirical criteria for interval-scale treatment along five dimensions.

### Granularity

The number of unique values produced by each score across the analytic sample. Reported as raw count and as a ratio (unique values / n). A higher ratio supports treatment as a quasi-continuous variable.

### Linearity of the logit (RCS)

Restricted cubic splines (RCS) with 4 knots placed at the 5th, 35th, 65th, and 95th percentiles of the score distribution [4]. For each binary outcome (HAP, mortality, FOIS ≤ 3), we fitted a logistic model with the score as a continuous RCS predictor and tested non-linearity via the likelihood-ratio test comparing the full RCS specification to a model with only the linear term. A non-significant non-linearity test (p > 0.05) supports interval-scale use.

### Information loss (AIC, continuous vs. quintile)

For each binary outcome, we compared the AIC of two logistic models: one with FDI-C as a continuous predictor, the other with FDI-C categorized into quintiles. ΔAIC = AIC(quintiles) − AIC(continuous); ΔAIC > 0 indicates that the continuous specification is preferred. Larger positive ΔAIC indicates greater information loss from categorization.

### Calibration

Observed outcome rates were computed across FDI-C deciles and plotted descriptively. Calibration was assessed visually for monotonicity and the presence of plateaus or transition zones (relevant for the mortality analysis; see Table 7).

### Construct validity against ordinal FOIS

Two analyses: (1) Spearman correlation between FDI-C and the full ordinal FOIS scale (1–7); (2) proportional-odds regression with FDI-C as an RCS predictor (4 knots) and ordinal FOIS as the outcome, with non-linearity tested as above. A linear proportional-odds RCS supports the use of FDI-C as a continuous predictor in clinical decision models.

## Online Resource 11 — Spearman-Brown derivation of FDI inter-rater reliability

Because FDI is computed deterministically from PAS and Yale ratings without additional rater-dependent judgments, its composite IRR was estimated using the **Spearman-Brown prophecy formula** [5]:

ρ_k = (k × ρ₁) / (1 + (k − 1) × ρ₁)

where ρ₁ is the single-consistency IRR and k is the number of consistencies tested.

### Single-consistency IRR sources

- **FDI-S** uses ρ₁ = 0.85, representative of PAS IRR in FEES [6, 7].
- **FDI-E** uses ρ₁ = 0.751, based on the lowest published Yale Pharyngeal Residue IRR [8, 9].

### Worked examples

| k (consistencies tested) | FDI-S projected IRR (ρ₁ = 0.85) | FDI-E projected IRR (ρ₁ = 0.751) |
| --- | --- | --- |
| 1 | 0.85 | 0.751 |
| 2 | 0.92 | 0.86 |
| 3 | 0.94 | 0.90 |
| 4 | 0.96 | 0.92 |

### Boundedness argument for FDI-C

Because PAS IRR exceeds Yale IRR at every k, the FDI-S component is at every k at least as reliable as FDI-E. The FDI-C composite is itself a deterministic average of FDI-S and FDI-E, and the reliability of an average of two correlated components is bounded from below by the reliability of the less reliable component (here, FDI-E). Therefore, conservatively:

ρ(FDI-C) ≥ ρ(FDI-E) = 0.86 (k = 2), 0.90 (k = 3), 0.92 (k = 4)

These lower bounds compare favorably to published composite-FEES-score IRRs (DIGEST-FEES total: weighted κ ≈ 0.82–0.83), and the FDI-C achieves this without requiring the rater-judgment components (frequency classification, aspiration amount estimation, residue percentage) that DIGEST-FEES [10] depends on.

### Limitation

The Spearman-Brown estimate is a **projection** from published single-consistency IRRs at other centers and populations, not an empirical IRR computed on the present study's raters.

## Online Resource 12 — R analysis environment

All analyses were performed in R version 4.5.2 [11]. The following packages were used:

| Package | Use | Reference |
| --- | --- | --- |
| pROC | AUC estimation and DeLong comparisons | [12] |
| rms | Restricted cubic splines (RCS), proportional odds with RCS | [4] |
| MASS | Proportional odds regression (polr) | — (Venables & Ripley) |
| cobalt | Covariate balance diagnostics for IPW | — |
| ordinal | Cumulative link mixed models (CLMMs) for Cohort 1 consistency-specific PAS analyses | — |
| Base R stats | Logistic regression (glm), Spearman correlation (cor) | — |

Random seeds were set for the stratified 60/40 derivation/validation split (Online Resource 4) and for bootstrap confidence intervals in the weighting sensitivity analysis (Online Resource 3).

## References

[1] Gilbert, T, Neuburger, J, Kraindler, J, Keeble, E, Smith, P, Ariti, C, Arora, S, Street, A, Parker, S, Roberts, HC, Bardsley, M, Conroy, S (2018) Development and validation of a Hospital Frailty Risk Score focusing on older people in acute care settings using electronic hospital records: an observational study. The Lancet 391(10132) :1775–1782 https://doi.org/10.1016/S0140-6736(18)30668-8.

[2] Koch, D, Kutz, A, Haubitz, S, Baechli, C, Gregoriano, C, Conca, A, Volken, T, Schuetz, P, Mueller, B (2020) Association of functional status and hospital-acquired functional decline with 30-day outcomes in medical inpatients: A prospective cohort study. Appl. Nurs. Res. 54 :151274 https://doi.org/10.1016/j.apnr.2020.151274.

[3] Koch, D, Schuetz, P, Haubitz, S, Kutz, A, Mueller, B, Weber, H, Regez, K, Conca, A, for the Triage Study Group (2019) Improving the post-acute care discharge score (PACD) by adding patients’ self-care abilities: A prospective cohort study. PLoS One 14(3) :e0214194 https://doi.org/10.1371/journal.pone.0214194.

[4] Harrell , FE (2015) Regression Modeling Strategies: With Applications to Linear Models, Logistic and Ordinal Regression, and Survival Analysis. Springer International Publishing, Cham.

[5] Nunnally, JC, Bernstein, IH (1994) Psychometric theory. McGraw-Hill, New York.

[6] Borders, JC, Brates, D (2020) Use of the Penetration-Aspiration Scale in Dysphagia Research: A Systematic Review. Dysphagia 35(4) :583–597 https://doi.org/10.1007/s00455-019-10064-3.

[7] Butler, SG, Markley, L, Sanders, B, Stuart, A (2015) Reliability of the penetration aspiration scale with flexible endoscopic evaluation of swallowing. Ann Otol Rhinol Laryngol 124(6) :480–483 https://doi.org/10.1177/0003489414566267.

[8] Neubauer, PD, Rademaker, AW, Leder, SB (2015) The Yale Pharyngeal Residue Severity Rating Scale: An Anatomically Defined and Image-Based Tool. Dysphagia 30(5) :521–528 https://doi.org/10.1007/s00455-015-9631-4.

[9] Pisegna, JM, Borders, JC, Kaneoka, A, Coster, WJ, Leonard, R, Langmore, SE (2018) Reliability of Untrained and Experienced Raters on FEES: Rating Overall Residue is a Simple Task. Dysphagia 33(5) :645–654 https://doi.org/10.1007/s00455-018-9883-x.

[10] Starmer, HM, Arrese, L, Langmore, S, Ma, Y, Murray, J, Patterson, J, Pisegna, J, Roe, J, Tabor-Gray, L, Hutcheson, K (2021) Adaptation and Validation of the Dynamic Imaging Grade of Swallowing Toxicity for Flexible Endoscopic Evaluation of Swallowing: DIGEST-FEES. J. Speech Lang. Hear. Res. 64(6) :1802–1810 https://doi.org/10.1044/2021_JSLHR-21-00014.

[11] R Core Team (2021) R: A Language and Environment for Statistical Computing.

[12] Robin, X, Turck, N, Hainard, A, Tiberti, N, Lisacek, F, Sanchez, J-C, Müller, M (2011) pROC: an open-source package for R and S+ to analyze and compare ROC curves. BMC Bioinformatics 12(1) :77 https://doi.org/10.1186/1471-2105-12-77.
